# Supplementary material for: Microbiome Responses to Oral Fecal Microbiota Transplantation in a Cohort of Domestic Dogs
Source: Vet Sci. 2024 Jan 19;11(1):42. doi: 10.3390/vetsci11010042 (PMC10821121; doi:10.3390/vetsci11010042)
Supplement: Supplementary file 1 [file vetsci-11-00042-s001.zip › vetsci-2738834-supplementary/Supplementary Figures.pdf]

SUPPLEMENTARY FIGURES

Microbiome responses to oral fecal microbiota transplantation in a cohort of domestic dogs

Connie A. Rojas[1], Zhandra Entrolezo[1], Jessica K. Jarett[1], Guillaume Jospin[1], Alex Martin[1], Holly H. Ganz[1]

[1] AnimalBiome, Oakland, CA, USA

Figure S1. Fecal microbiome composition before and after FMT for the fifty-four FMT recipients. Plots showing the relative frequencies of 16S rRNA gene sequences assigned to bacterial genera with mean relative abundances > 1.65%, while all other genera are clumped into an "Other" category.

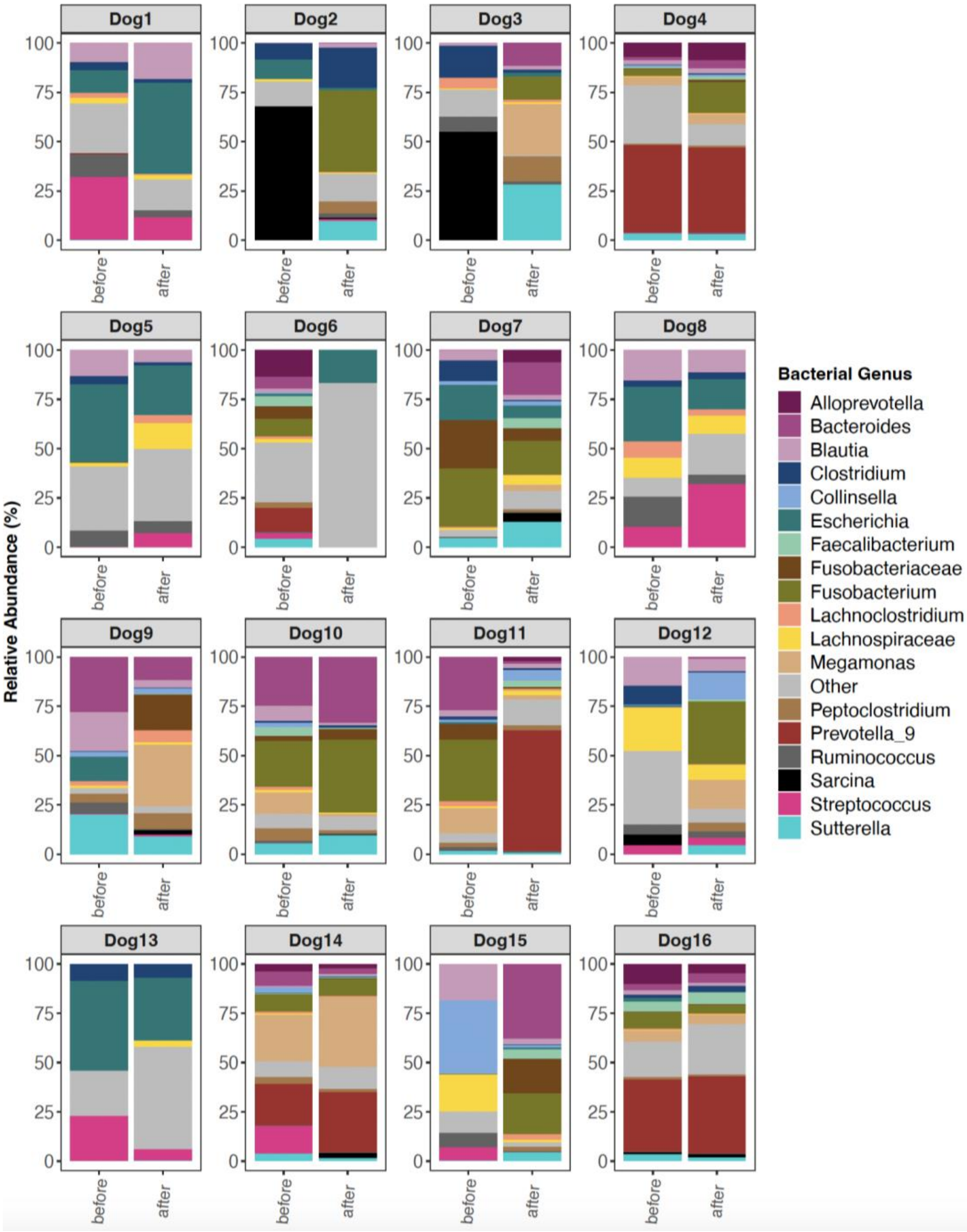

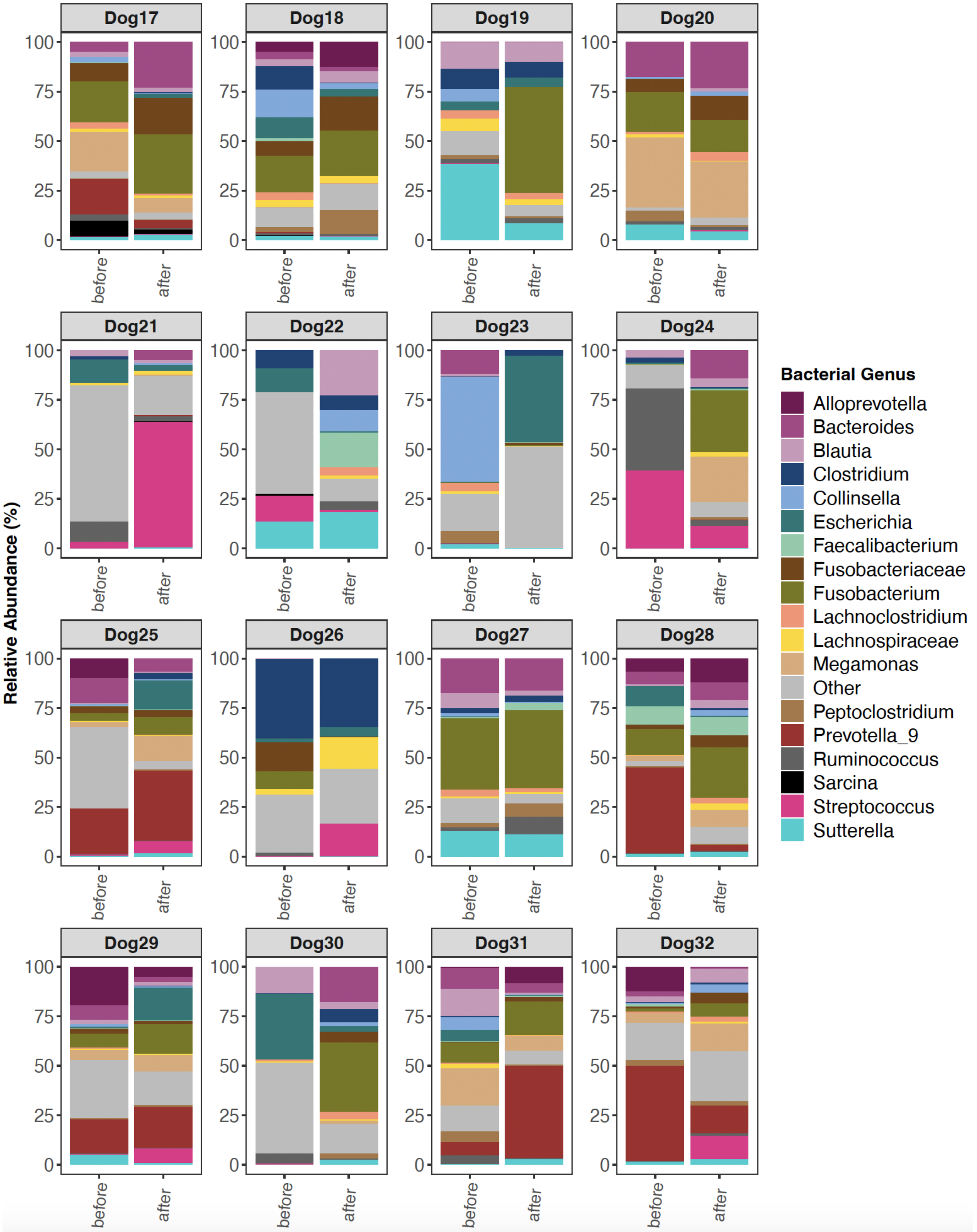
Dog17
Dog18
Dog19
Dog20
Dog21
Dog22
Dog23
Dog24
Dog25
Dog26
Dog27
Dog28
Dog29
Dog30
Dog31
Dog32
Relative Abundance (%)
0
25
50
75
100
before
after
Bacterial Genus
Alloprevotella
Bacteroides
Blautia
Clostridium
Collinsella
Escherichia
Faecalibacterium
Fusobacteriaceae
Fusobacterium
Lachnoclostridium
Lachnospiraceae
Megamonas
Other
Peptoclostridium
Prevotella_9
Ruminococcus
Sarcina
Streptococcus
Sutterella

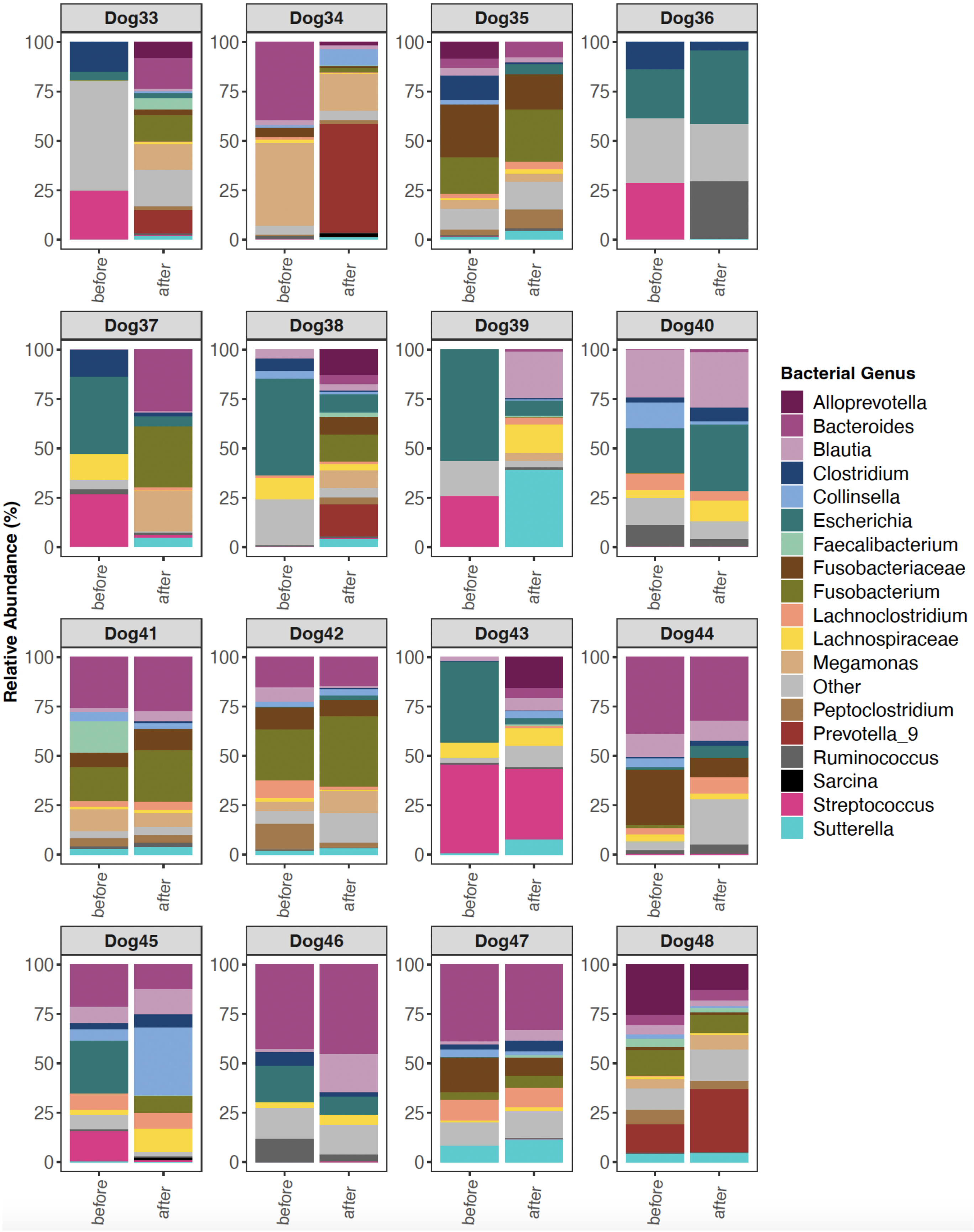
Dog33
Dog34
Dog35
Dog36
Dog37
Dog38
Dog39
Dog40
Dog41
Dog42
Dog43
Dog44
Dog45
Dog46
Dog47
Dog48
100
75
50
25
0
before
after
Relative Abundance (%)
Bacterial Genus
Alloprevotella
Bacteroides
Blautia
Clostridium
Collinsella
Escherichia
Faecalibacterium
Fusobacteriaceae
Fusobacterium
Lachnoclostridium
Lachnospiraceae
Megamonas
Other
Peptoclostridium
Prevotella_9
Ruminococcus
Sarcina
Streptococcus
Sutterella

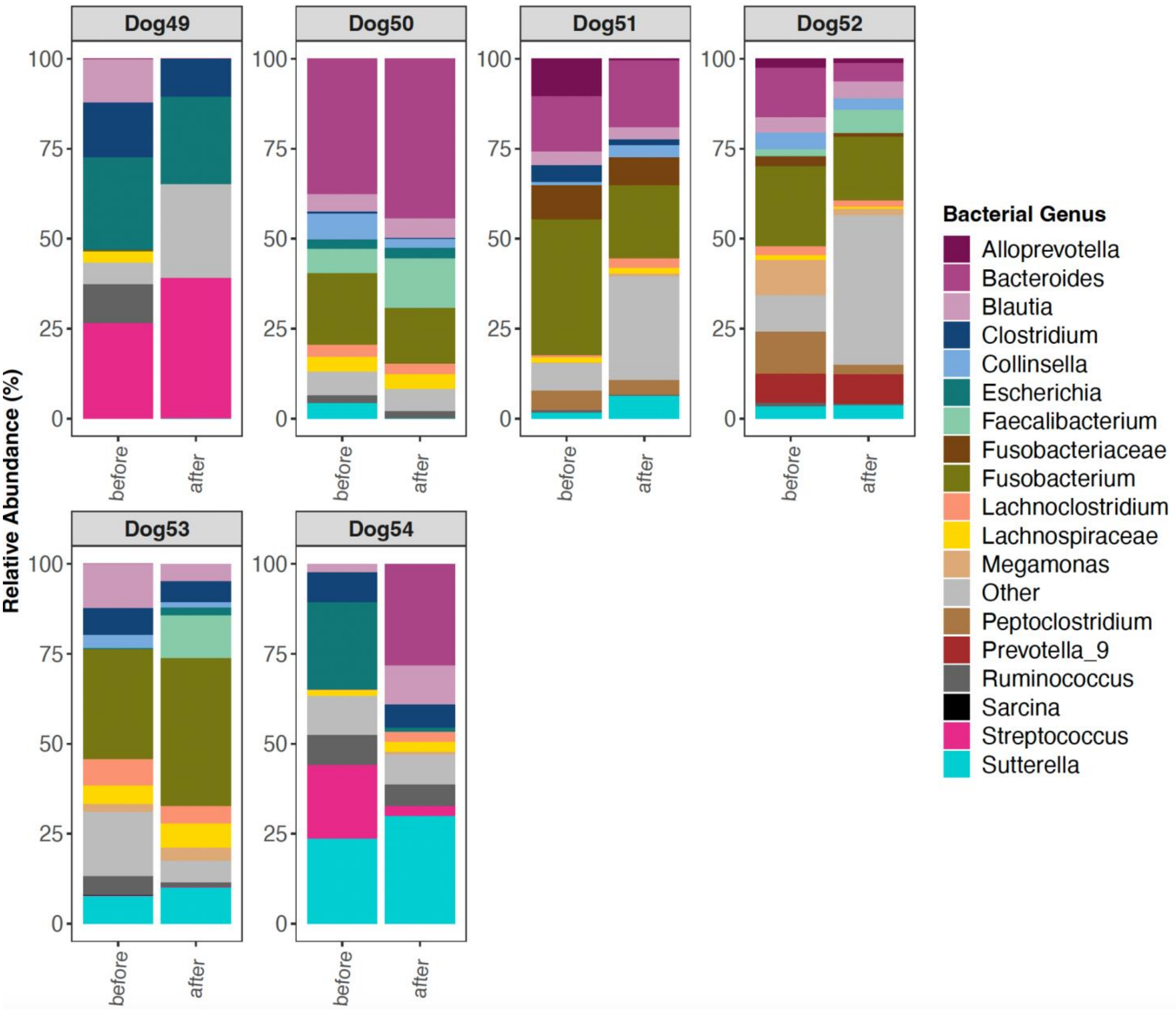
Dog49
Dog50
Dog51
Dog52
Dog53
Dog54
Relative Abundance (%)
100
75
50
25
0
before
after
Bacterial Genus
Alloprevotella
Bacteroides
Blautia
Clostridium
Collinsella
Escherichia
Faecalibacterium
Fusobacteriaceae
Fusobacterium
Lachnoclostridium
Lachnospiraceae
Megamonas
Other
Peptoclostridium
Prevotella_9
Ruminococcus
Sarcina
Streptococcus
Sutterella
